# K^+^ Channel Regulator KCR1 Suppresses Heart Rhythm by Modulating the Pacemaker Current I_f_

**DOI:** 10.1371/journal.pone.0001511

**Published:** 2008-01-30

**Authors:** Guido Michels, Fikret Er, Ismail F. Khan, Jeannette Endres-Becker, Mathias C. Brandt, Natig Gassanov, David C. Johns, Uta C. Hoppe

**Affiliations:** 1 Department of Internal Medicine III, University of Cologne, Cologne, Germany; 2 Gene Vector Center, The Johns Hopkins University, Baltimore, Maryland, United States of America; 3 Center for Molecular Medicine, University of Cologne (CMMC), Cologne, Germany; Vrije Universiteit Amsterdam, Netherlands

## Abstract

Hyperpolarization-activated, cyclic nucleotide sensitive (HCN) channels underlie the pacemaker current I_f_, which plays an essential role in spontaneous cardiac activity. HCN channel subunits (HCN1-4) are believed to be modulated by additional regulatory proteins, which still have to be identified. Using biochemistry, molecularbiology and electrophysiology methods we demonstrate a protein-protein interaction between HCN2 and the K^+^ channel regulator protein 1, named KCR1. In coimmunoprecipitation experiments we show that KCR1 and HCN2 proteins are able to associate. Heterologously expressed HCN2 whole-cell current density was significantly decreased by KCR1. KCR1 profoundly suppressed I_HCN2_ single-channel activity, indicating a functional interaction between KCR1 and the HCN2 channel subunit. Endogenous KCR1 expression could be detected in adult and neonatal rat ventriculocytes. Adenoviral-mediated overexpression of KCR1 in rat cardiomyocytes (i) reduced I_f_ whole-cell currents, (ii) suppressed most single-channel gating parameters, (iii) altered the activation kinetics, (iv) suppressed spontaneous action potential activity, and (v) the beating rate. More importantly, siRNA-based knock-down of endogenous KCR1 increased the native I_f_ current size and single-channel activity and accelerated spontaneous beating rate, supporting an inhibitory action of endogenous KCR1 on native I_f_. Our observations demonstrate for the first time that KCR1 modulates I_HCN2_/I_f_ channel gating and indicate that KCR1 serves as a regulator of cardiac automaticity.

## Introduction

Hyperpolarization-activated cation channels are found in a variety of cardiac cells and neurons [1]–[3]. These channels activate in response to hyperpolarization to generate an inward current termed I_f_ (“funny”) in cardiac cells, I_h_ (“hyperpolarization”-activated) in neurons, or I_q_ (“queer”). I_f_ has been proposed to contribute to pacemaker depolarization which generates rhythmic activity in spontaneously active cardiac cells [4], [5] and neurons [6], [7]. A family of four homologous hyperpolarization-activated, cyclic nucleotide-gated ion channel subunits (HCN1-4) have been identified [8]–[11]. In heterologous expression all HCN channels give rise to a hyperpolarization-activated inward current with similar but not identical characteristics compared to native I_f_ [8], [9], [12]. These observations suggest that HCN channel function is likely to be modulated by regulatory proteins and β-subunits in myocardial tissue.

The K^+^ channel regulator 1 (KCR1), originally cloned from rat cerebellum, is a plasma membrane-associated protein with 12 putative transmembrane regions which is also expressed in rat cerebrum, and in rat and human heart [13], [14]. The KCR1 protein can associate with rat ether-à-go-go (EAG) and human ether-à-go-go related (HERG) channel subunits [13]–[15]. Given the structural similarity and sequence analogy of HERG and HCN genes, we speculated that KCR1 might also interact with HCN channel subunits [8], [9], [12]. Therefore, we evaluated whether KCR1 and HCN2 proteins can associate. Secondly, we aimed to determine any possible functional modulation of I_HCN2_ and native I_f_ current characteristics by KCR1 in electrophysiological studies. Our results show that KCR1 and HCN2 proteins interact and demonstrate that KCR1 profoundly alters I_HCN2_ and I_f_ gating properties. Furthermore, KCR1 suppressed spontaneous rhythmicity in cardiocytes. Thus, our observations indicate that KCR1 serves as a regulatory protein of native I_f_.

## Results

### KCR1 and HCN2 associate in a protein complex

**Figure 1:**
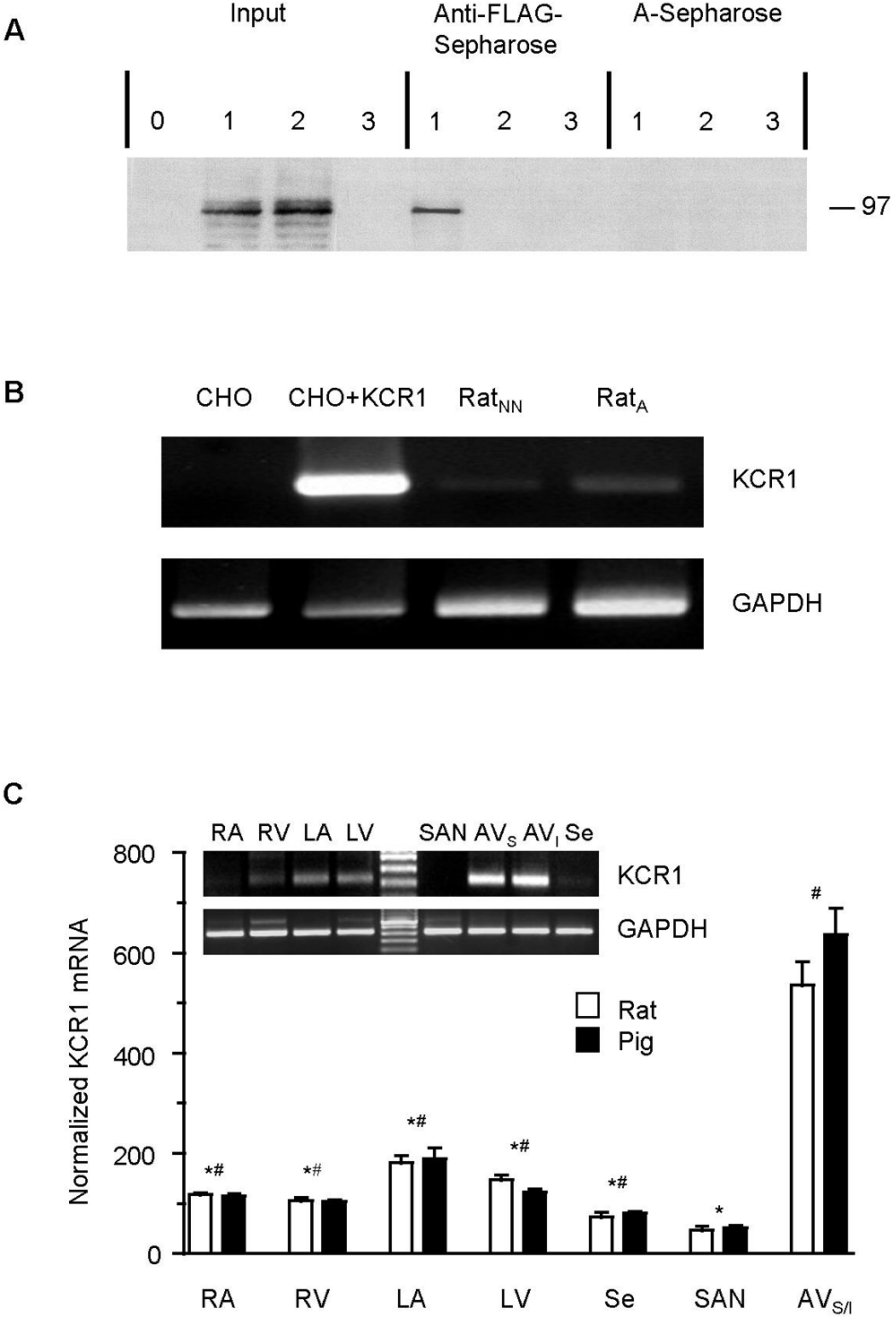
Analysis of protein interaction between HCN2 and KCR1 and expression of KCR1 in various cell types. (A) KCR1 and HCN2 can associate in protein complexes. Coimmunoprecipitation of HCN2 and KCR1 from mammalian cell extracts. Protein extracts from cells transfected with pCFLAG^3^-KCR1 (lane 0), HCN2 plus pCFLAG^3^-KCR1 (lanes 1), HCN2 plus p3xFLAG-CMV (lanes 2) or non-transfected cells (lanes 3) assayed by Western blot using anti-HCN2 antibody. Input: input lysates were blotted to show that HCN2 protein was successfully produced and detected. Anti-FLAG-Sepharose: 700 µg of total lysate immunoprecipitated with anti-FLAG-Sepharose shows that HCN2 and KCR1 (pCFLAG^3^-KCR1) can be coimmunoprecipitated (lane 1). A-Sepharose: mock immunoprecipitation using protein A-Sepharose beads unlinked to FLAG-antibody to exclude any unspecific antibody binding. (B) KCR1 message (218 bp) can be detected in all cell types tested, except for non-transfected CHO cells (upper panel). Representative electrophoresis gel illustrating results obtained in single-cell RT-PCR experiments (as described in Methods) with three different cells types. CHO: Non-transfected Chinese hamster ovary cells; CHO+KCR1: CHO cells transfected with KCR1 cDNA; Rat_NN_: Neonatal rat cardiomyocytes; Rat_A_: Adult rat cardiomyocytes. GAPDH mRNA (318 bp) was used as a control and is found in all cell types tested, including non-transfected, KCR1-negative CHO cells (lower panel). Negative controls did not include RNA or reverse transcriptase and gave no amplicons (not shown). (C) Regional differences in expression of KCR1 in adult rat and pig heart. KCR1 mRNA levels determined by quantitative real-time PCR (qPCR; n = 3–4). RA = right atrium, RV = right ventricular free wall, LA = left atrium, LV = left ventricular free wall, Se = septum, SAN = sinoatrial node, AV = atrioventricular node. Values are mean±SEM. *p<0.05 vs. AV node, ^#^p<0.05 vs. SAN. Upper panel: Representative electrophoresis gel obtained from qPCR products of different cardiac regions (the AV-node region was divided in: AV_S_ = superior part and AV_I_ = inferior part), samples were loaded and normalized to GAPDH.

To determine whether HCN and KCR1 gene products can form a protein complex, we prepared protein extracts from CHO cells cotransfected with KCR1 cDNA incorporating triple FLAG tags at the 5′ end (pCFLAG^3^-KCR1) and HCN2 cDNA. Control cells were transfected with pCFLAG^3^-KCR1 alone or cotransfected with HCN2 and the empty FLAG-epitope containing vector. Input lysates were assayed in Western blots using an anti-HCN2 antibody to show successful production and detection of the HCN2 protein (Figure 1A). In addition, cell lysates were immunoprecipitated with anti-FLAG-Sepharose and then blotted using the anti-HCN2 antibody. Indeed, a band with the expected molecular mass of HCN2 was detected by the anti-HCN2 antibody in cells cotransfected with HCN2 and pCFLAG^3^-KCR1, whereas it could not be coimmunoprecipitated from extracts containing pCFLAG^3^-KCR1 alone or HCN2 and the empty FLAG-epitope containing vector (Figure 1A). These results indicate that HCN2 and KCR1 associate in protein complexes in mammalian cells, while excluding any unspecific detection of KCR1 by the anti-HCN2 antibody and any unspecific coimmunoprecipitation of the FLAG-epitope and HCN2. In addition, unspecific coimmunoprecipitation by the anti-FLAG-Sepharose could be excluded by mock immunoprecipitation with normal A-Sepharose (Figure 1A).

### KCR1 reduces HCN2 current size and profoundly modulates HCN2 channel gating

**Figure 2:**
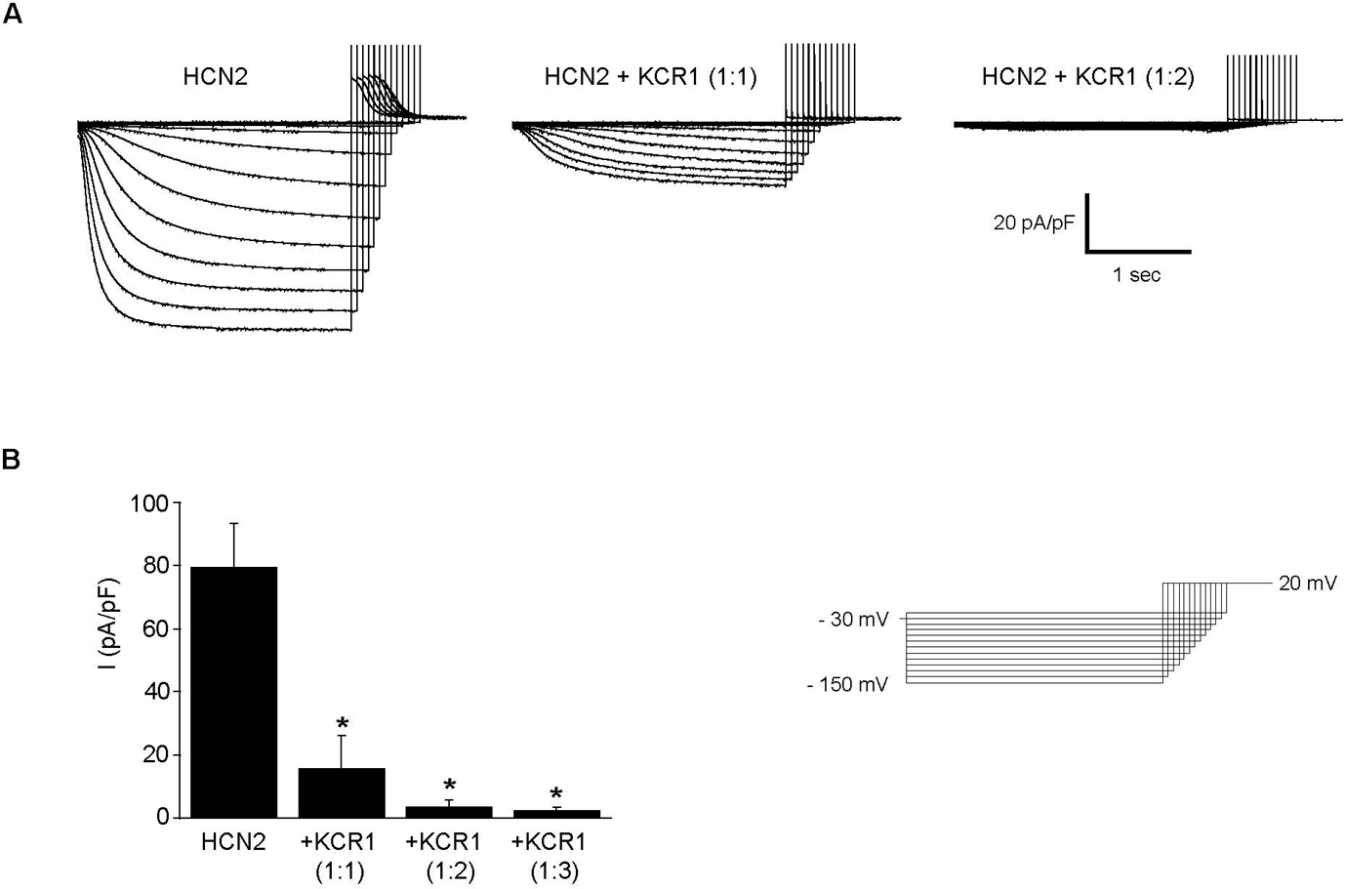
KCR1 suppresses recombinant I_HCN2_ current density. (A) Representative HCN2 whole-cell currents in absence and presence of KCR1. (B) Mean current densities of heterologously expressed HCN2 alone or together with KCR1 demonstrate that KCR1 significantly (_*_, p<0.001) reduced current density of recombinant I_HCN2_. For data see text.

To evaluate a possible functional interaction of HCN2 and KCR1, the effect of KCR1 on I_HCN2_ was analysed. Whole-cell I_HCN2_ currents were recorded from CHO cells transfected with HCN2 (0.25 µg/well) alone or together with KCR1 (ratio 1∶1, 1∶2 or 1∶3; total cDNA amount adjusted to 1 µg/well with the unrelated channel subunit Kv1.3AYA in all experiments). RT-PCR revealed no detectable KCR1 in non-transfected CHO cells (Figure 1B). Representative current recordings (Figure 2A) and mean current densities (Figure 2B) show that I_HCN2_ (79.6±13.9 pA/pF at −130 mV, n = 9) was significantly decreased by KCR1 (15.8±10.4 pA/pF, ratio 1∶1, n = 14; 3.6±2.1 pA/pF, ratio 1∶2, n = 10; 2.5±0.9 pA/pF, ratio 1∶3, n = 17; p<0.001). Moreover, KCR1 significantly shifted half-maximal activation of I_HCN2_ (−102.0±2.1 mV) to more negative potentials (−109.8±2.0 mV for HCN2+KCR1, ratio 1∶1; p = 0.048; n = 12).

**Figure 3:**
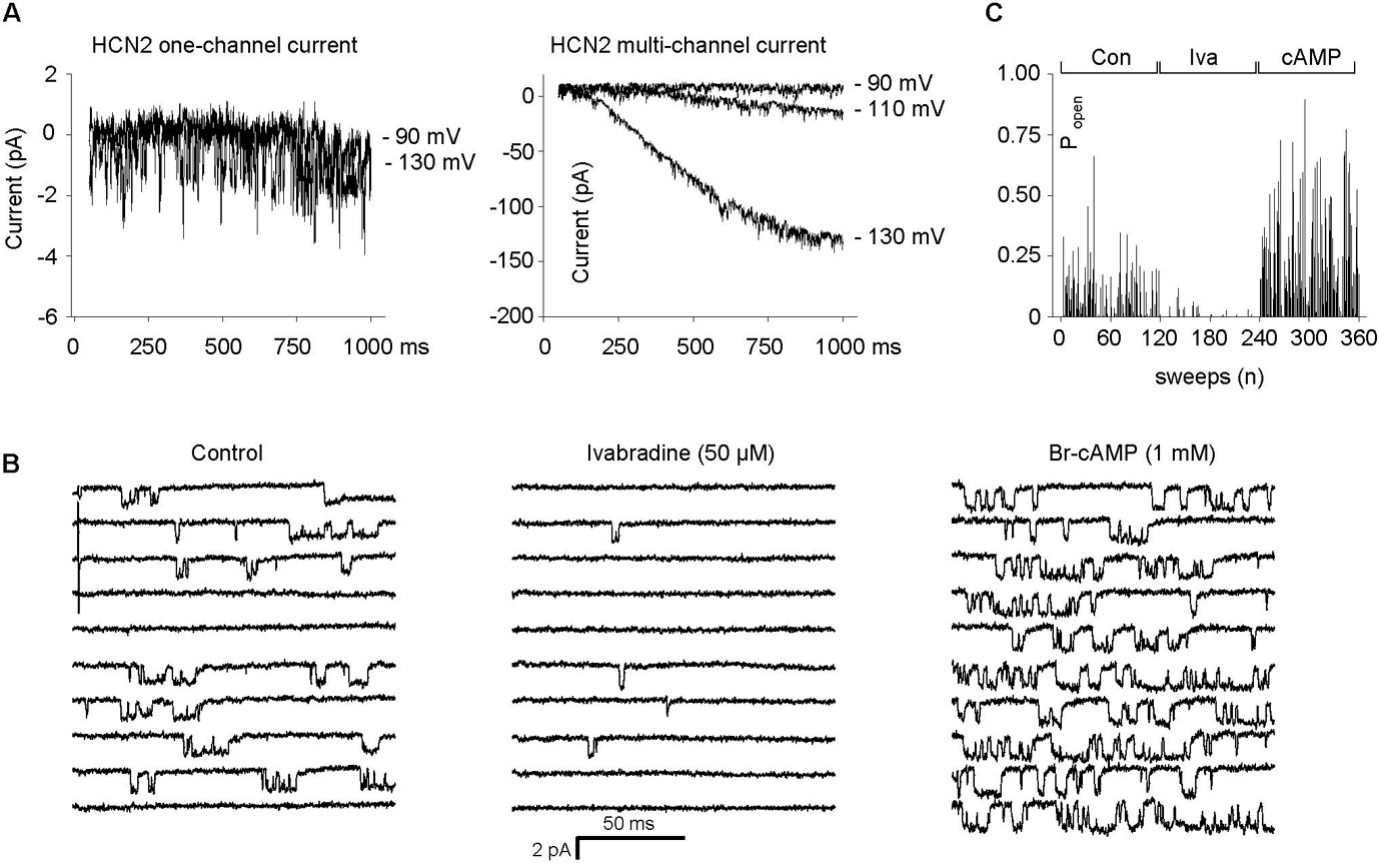
Electrophysiological and pharmacological properties of I_HCN_/I_f_ on single-channel level from inside-out recordings. (A) Representative HCN2 single-channel currents (sampling frequency: 5 kHz, corner frequency: 1 kHz) of inside-out recordings from one- and multi-channel (n∼50 channels) patches at different test-potentials (−90 to −130 mV). (B) Pharmacological characteristics of HCN2 single-channels (test potential: −90 mV, holding potential: −35 mV). Ivabradine (50 µM) blocks HCN2 single-channel current during repetitive activation/deactivation steps (−90 mV, 150 ms/+10 mV, 600 ms). The observations that ivabradine significantly reduced the open probability (25.8±7.07% vs.6.08±3.45%, n = 4, p<0.05), the mean open time (1.03±0.12 ms vs. 0.61±0.16 ms, n = 4, p<0.05) and the availability (75.9±10.1% vs. 25.3±6.98%, n = 4, p<0.05) suggests an open-channel blockade by a fast and a slow gating mechanism. cAMP induced an increase of the channel activity (for data, see text). The data were sampled at 10 kHz and filtered at 2 kHz. (C) Effect of ivabradine (Iva) and cAMP on single-channel activity. Open probability (P_open_) decreased after ivabradine (50 µM) and increased after cAMP (1 mM) application, respectively. Data recorded as in Figure 3B.

To further confirm a functional modulation of HCN2 channel properties by KCR1 additional single-channel recordings were performed. Available data about HCN single-channel properties vary depending on cell-type and recording technique [16]–[20]. While we previously already showed characteristic stimulation of single HCN2 channels by forskolin [16], we now further proved that the channel openings in our experiments were produced by HCN channels demonstrating (i) a time- and voltage-dependent kinetic similar to macroscopic I_HCN_/I_f_ current from HCN2 multi-channel recordings, which allows in contrast to “pure” one-channel recording cooperative HCN channel gating (Figure 3A), (ii) a HCN2 single-channel conductance of these multi-channel patches (24.0±4.62 pS, n = 3) comparable to our cell-attached recordings (considering the symmetrical K^+^-solution in inside-out recordings) (Figure 3A), (iii) a typical increase of the open probability upon cAMP application in inside-out recordings (control: 24.7±11.1% vs. Br-cAMP 1 mM: 51.0±14.1%, n = 4, p<0.05) (Figure 3B and 3C), and (iv) a reduction of HCN2 open probability by the specific I_f_ inhibitor ivabradine (50 µM) (25.8±7.07% vs. 6.08±3.45%, n = 4, p<0.05) (Figure 3B and 3C).

**Figure 4:**
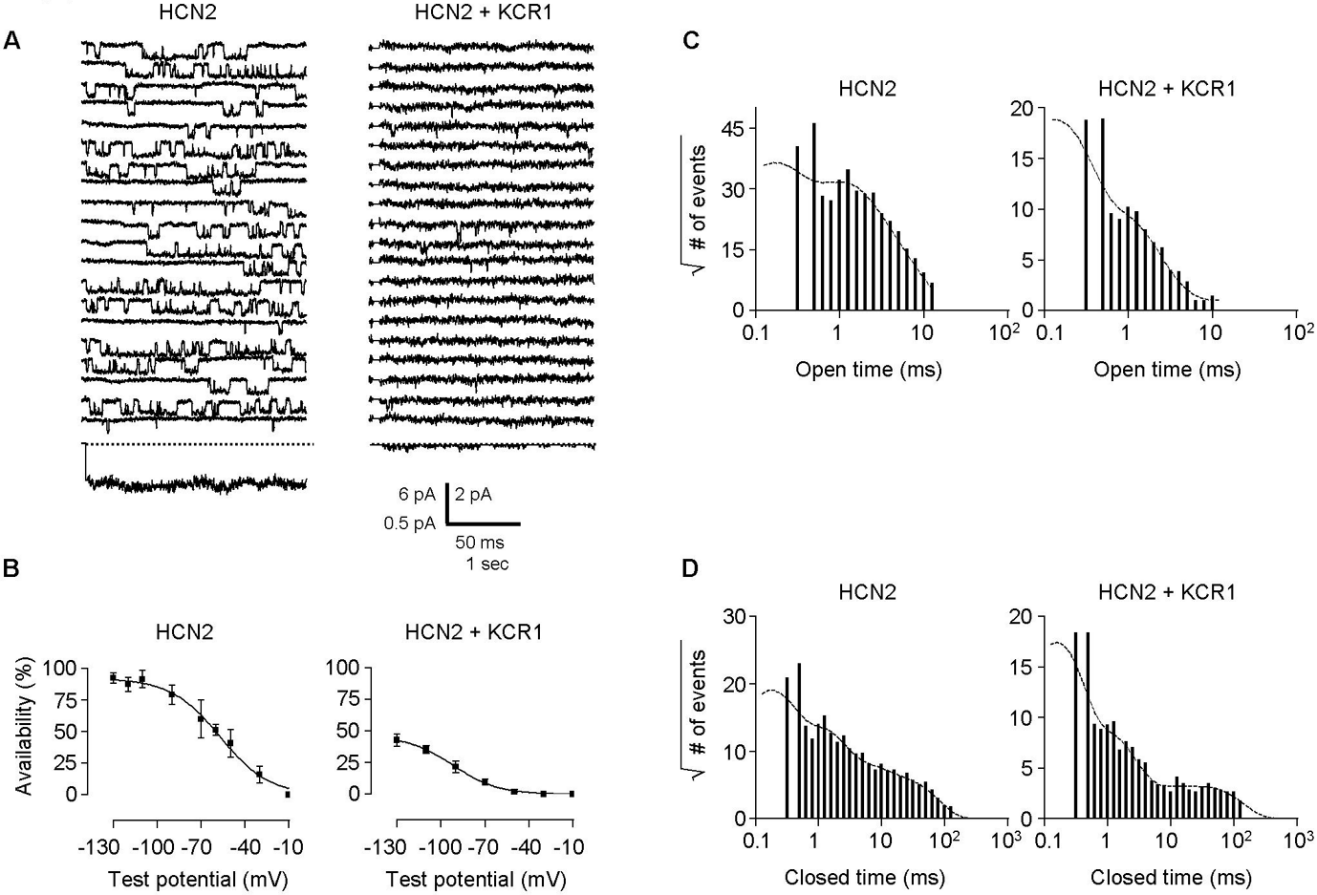
Effect of KCR1 on single recombinant HCN2 channel gating in one-channel patches. (A) Comparison of single recombinant HCN2 channels transfected in CHO cells alone (left) and with KCR1 (right). Middle, 20 consecutive single traces of each channel without and with KCR1. Single channels were hyperpolarized at continuous pulse mode for a total duration of 3 s (20×150 ms sweeps), with a holding potential of −35 mV and a test potential of −90 mV. Bottom, ensemble average current of one consecutive sweep of 3 s pulse duration. Scale bars, 50 ms, 6 pA (unitary current traces) for HCN2 alone (left) and 2 pA when co-transfected with KCR1 (right), or 1 s, 0.5 pA (ensemble average current) for HCN2 and HCN2+KCR1. (B) KCR1 significantly shifted I_HCN2_ activation to more negative potentials. Channel activation was measured by the parameter availability, plotted against the test potential and then determined by using the Boltzmann function. For data see text. (C) Open-time histograms: KCR1 reduced the number of HCN2 open states. Number of open events (square root) were plotted against the logarithmically binned open time durations for HCN2 alone and HCN2+KCR1 (pooled one- and multi [n≤3]-channel experiments). (D) Closed-time histograms: KCR1 did not affect the number of HCN2 closed states. Number of closed events (square root) were plotted against the logarithmically binned closed time durations for HCN2 alone and HCN2+KCR1 (pooled one-channel experiments only).

Single-channel characteristics of cells cotransfected with HCN2 and KCR1 were distinct from cells expressing HCN2 alone. KCR1 profoundly reduced single-channel activity of I_HCN2_ (Figure 4A; Table 1). Moreover, KCR1 caused a hyperpolarization shift of the voltage of half-maximal I_HCN2_ activation (V_0.5_ for I_HCN2_ and I_HCN2+KCR1(1:2)_ was −58.5±4.8 mV, n = 6, vs. −89.7±4.9 mV, n = 5, p = 0.001, with the slope factor k being unchanged: −15.8±3.0 mV and −12.4±2.7 mV, respectively; Figure 4B).

**Table 1:** Gating of single recombinant HCN2 and KCR1-cotransfected I_HCN2_

Parameter	HCN2 control	HCN2+KCR1 (1∶1)	HCN2+KCR1 (1∶2)
Open probability (%)	32.6±8.79	23.4±13.14	0.99±0.32*
Availability (%)	78.5±6.10	54.6±20.48	22.9±4.01*
Mean open time (ms)	1.15±0.15	0.76±0.16	0.44±0.06*
Mean closed time (ms)	2.94±0.45	1.23±0.44*	2.78±0.61
Mean first latency (ms)	37.2±6.27	38.0±17.14	42.9±5.94
Amplitude (pA)	−2.16±0.15	−0.86±0.06*	−0.68±0.01*
Conductance (pS)	34.6±2.431)	12.3±1.44*	8.39±0.63*
I_peak_ (fA)	809±170	398±204	43±14*
number of experiments	10 (6)	4 (4)	9 (4)

Single-channel parameters of HCN2 (control) and KCR1-cotransfected I_HCN2_ channels in CHO-cells. Holding potential −35 mV, test potential −90 mV. I_peak_ was measured from ensemble average currents. For closed time and latency analysis, only experiments containing just one detected open level were used for calculation. Numbers of experiments given in parentheses indicate number of experiments with only one channel in the patch. Pooled data are presented as mean±SEM.

* p<0.05 vs. control.

1) in this case n = 12 experiments were taken for conductance calculation.

We recently revealed an allosteric multi-state gating model comprising at least four open and five closed states of single HCN2 channel recordings [16], [21]. In the present study KCR1 altered HCN2 channel open kinetics to only three open states (HCN2-τ_open _values [ms]: 0.13 [47.5%], 0.86 [33.5%], 2.24 [18.5%], 5.93 [0.5%] (n = 8) vs. HCN2+KCR1 (1∶1)-τ_open _values [ms]: 0.13 [54.7%], 0.87 [38.5%], 2.72 [6.8%] (n = 4) and HCN2+KCR1 (1∶2)-τ_open _values [ms]: 0.13 [77%], 0.69 [21.9%], 2.36 [1.1%] (n = 5)), while not significantly affecting the five closed states (HCN2-τ_closed _values [ms]: 0.15 [55.7%], 0.90 [28.4%], 3.36 [5.5%], 5.52 [4.1%], 21.0 [6.3%] (n = 5) vs. HCN2+KCR1 (1∶1)-τ_closed _values [ms]: 0.12 [64.9%], 0.55 [18.9%], 0.97 [12%], 2.96 [3.8%], 27.0 [0.4%] (n = 4) and HCN2+KCR1 (1:2)-τ_closed _values [ms]: 0.14 [76.3%], 0.83 [16.8%], 1.78 [3.1%], 7.80 [1.3%], 37.92 [2.5%] (n = 4) (Figure 4C and 4D).

### KCR1 is highly expressed in the atrioventricular-node

While functional interaction of HCN and KCR1 gene products in heterologous mammalian systems is interesting, it is even more important whether KCR1 is present in cardiac tissue and might also modulate native I_f_. To clarify this situation we chose neonatal and adult rat ventriculocytes. To omit any interference of non-cardiac cells, we performed reverse transcriptase PCR analysis of single neonatal and adult rat ventricular myocytes. Indeed, in both cell types the expected band could be detected (Figure 1B), demonstrating endogenous expression of KCR1 in native cardiac cells.

To analyze regional expression of KCR1, we determined KCR1 mRNA levels of freshly dissected samples from different parts of adult rat and pig hearts by quantitative real-time PCR. In both species we consistently obtained highest KCR1 expression in the atrioventricular node (rat: 536±48%, n = 3; pig: 637±52%, n = 3; p<0.05 vs. all other regions) and lowest levels of KCR1 in the sinus node (rat: 46.3±8.10%, n = 3; pig: 52.7±4.98%, n = 3; p<0.05 vs. all other regions) (Figure 1C).

### KCR1 overexpression suppresses current density and single-channel activity of native I_f_

**Figure 5:**
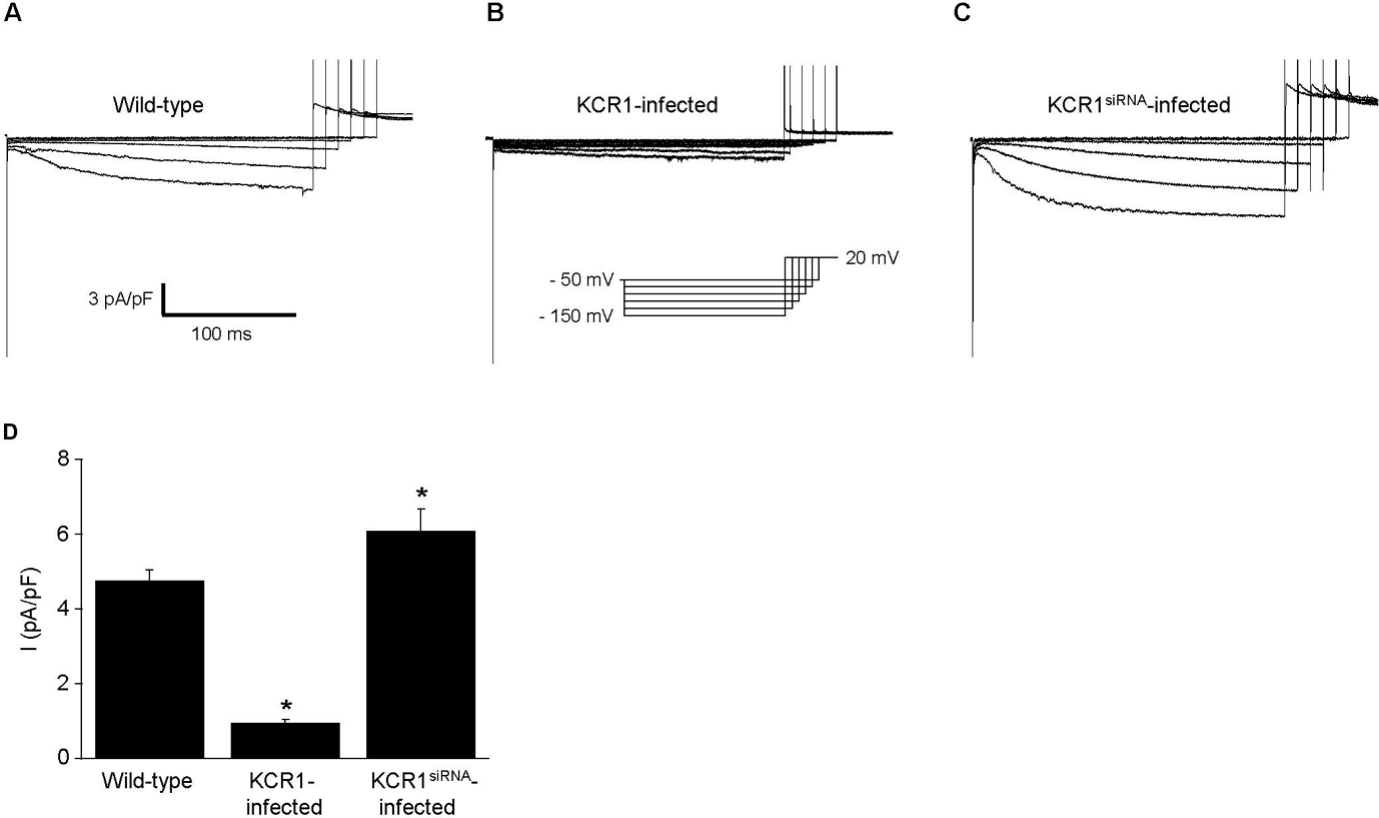
KCR1 reduces current size of native I_f_ in neonatal rat cardiomyocytes. (A–C) Original whole-cell recordings of I_f_ in neonatal rat cardiocytes: (A) control, (B) KCR1-infected and (C) KCR1^siRNA^-infected. (D) Mean current densities of I_f_ in neonatal cells show that KCR1 overexpression reduced I_f_, while suppression of endogenous KCR1 by KCR1^siRNA^ significantly increased native I_f _(_*_, p<0.001). For data see text.

Considering our results in heterologous expression, overexpression of a construct carrying KCR1 should reduce I_f_ current density in myocardium if HCN and KCR1 also interact in native tissue. In neonatal ventriculocytes I_f_ density was indeed markedly reduced from 4.7±0.3 pA/pF (n = 14) in control cells infected with adenoviral vectors expressing EGFP alone to 0.9±0.1 pA/pF (n = 9) in KCR1-infected cells when measured at −130 mV (p<0.001) (Figure 5A, 5B and 5D). Similarly, I_f_ current size in adult rat ventricular myocytes was significantly suppressed from 3.3±0.5 pA/pF (n = 11) in controls to 0.38±0.08 pA/pF (at −130 mV; n = 10) in KCR1-infected myocytes (p<0.001).

**Figure 6:**
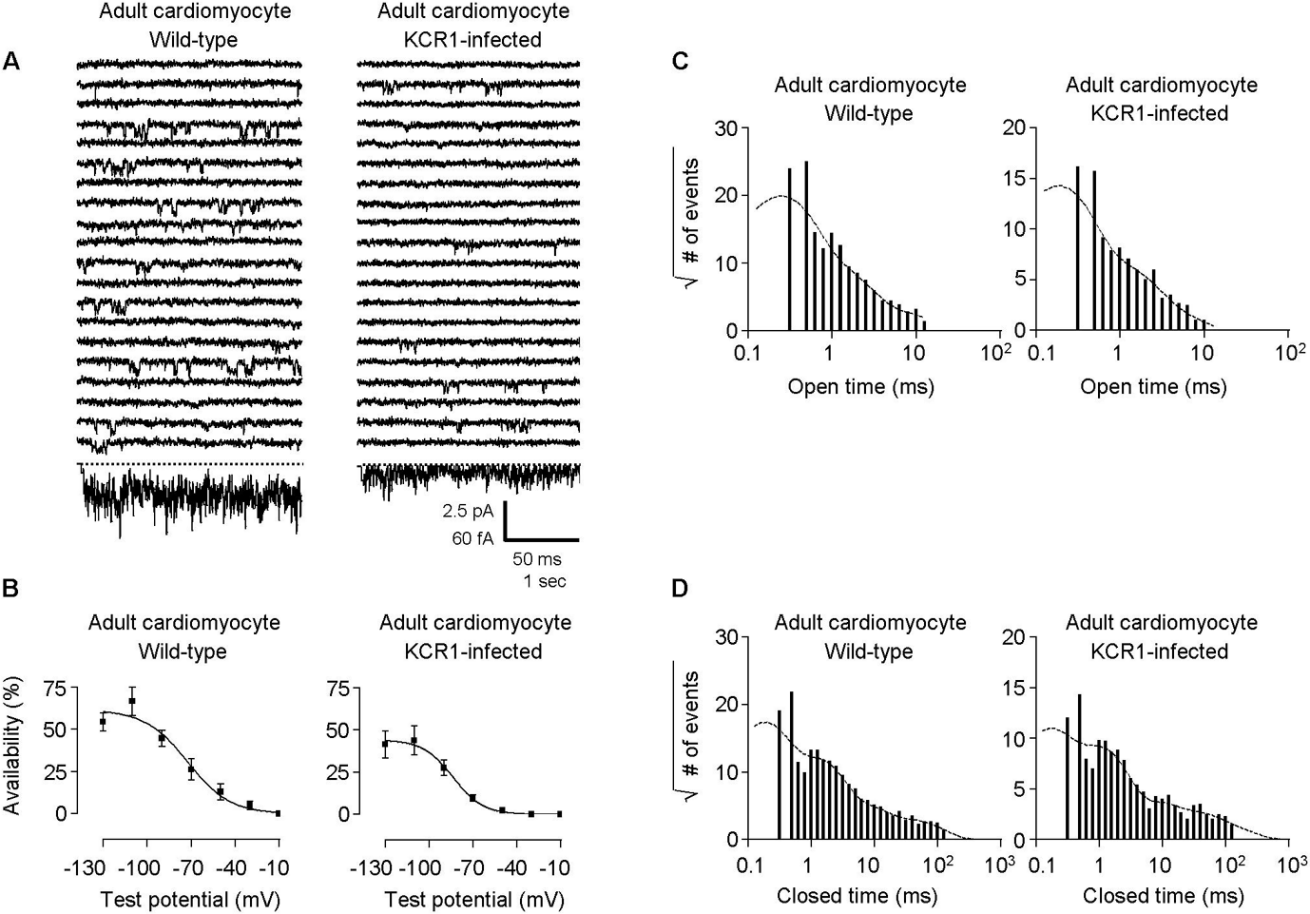
Effect of KCR1 on single native I_f_ channel gating in one-channel patches. (A) Comparison between single native I_f_ channels of control adult ventriculocytes (left) and KCR1-infected cells (right). Recording technique as in Figure 3. Scale bars, 50 ms, 2.5 pA for unitary current traces, and 1 s, 60 fA for ensemble average current. (B) KCR1 significantly shifted I_f_ activation to more negative potentials. Channel activation was measured by the parameter availability, plotted against the test potential and then determined by using the Boltzmann function. For data see text. (C) Open-time histograms: KCR1 exhibited no effect on the number of native I_f_ open states. Number of open events (square root) were plotted against the logarithmically binned open time durations for I_f_ alone and with exogenous KCR1 (pooled one- and multi [n≤3]-channel experiments). (D) Closed-time histograms: KCR1 did not affect the number of native I_f_ closed states. Number of closed events (square root) were plotted against the logarithmically binned closed time durations for I_f_ alone and with exogenous KCR1 (pooled one-channel experiments only).

**Figure 7:**
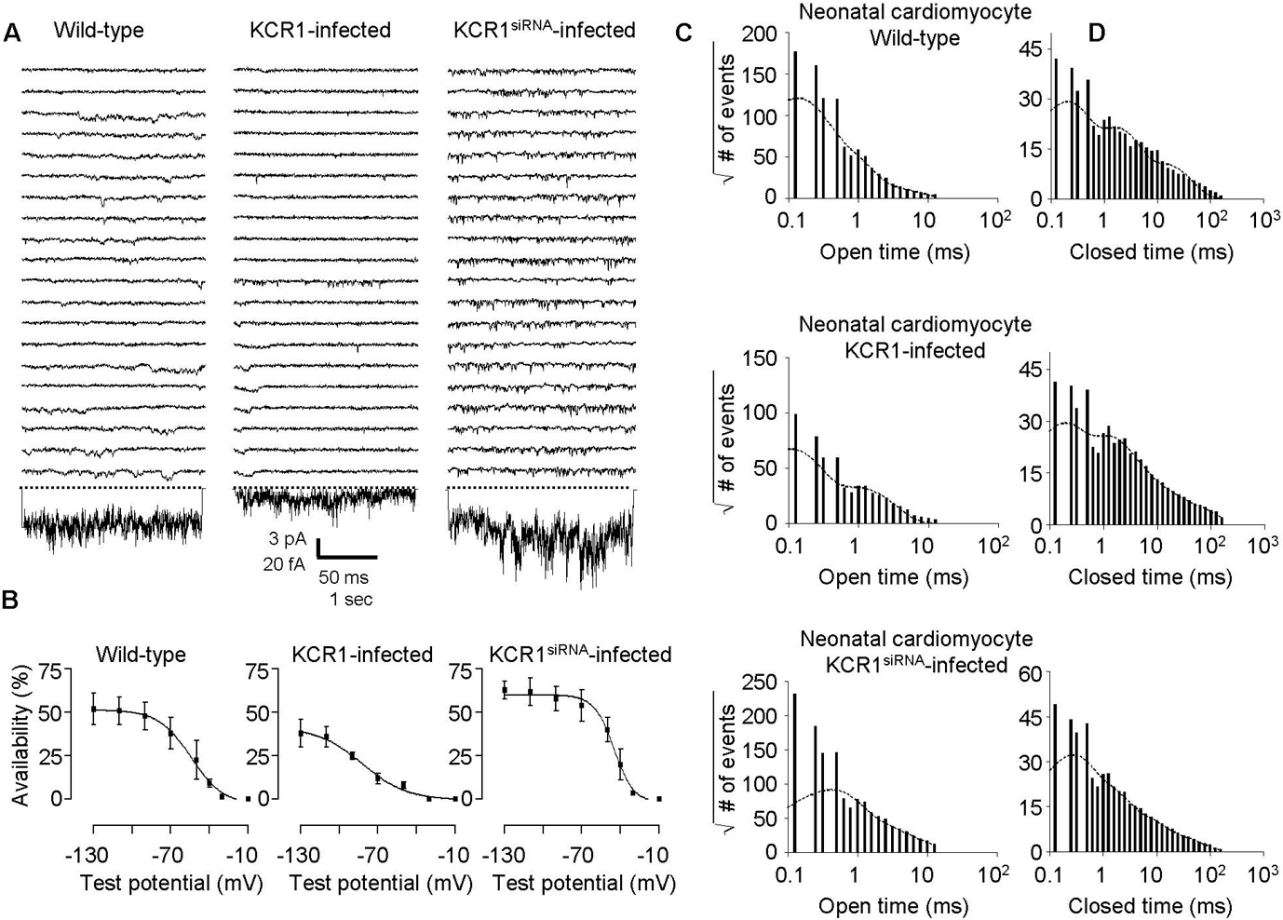
Effect of KCR1 and KCR1^siRNA^ on single native I_f_ channel gating in one-channel patches. (A) Comparison between single native I_f_ channels of control neonatal ventriculocytes (left), KCR1-infected (middle) and KCR1^siRNA^-infected cells (right). Recording technique as in Figure 3. Scale bars, 50 ms, 3 pA for unitary current traces, and 1 s, 20 fA for ensemble average current. (B) Enhanced expression and knock-down of KCR1 significantly shifted the half-maximal activation of I_f_ to more negative and more positive potentials, respectively. Channel activation was measured by the parameter availability, plotted against the test potential and then determined by using the Boltzmann function. For data see text. (C) Open-time histograms: KCR1 exhibited no effect on the number of native I_f_ open states, whereas KCR1^siRNA^ induced an increase of the number of open states. Number of open events (square root) were plotted against the logarithmically binned open time durations for I_f_ alone and with exogenous KCR1 or KCR1^siRNA^ (pooled one- and multi [n≤3]-channel experiments). (D) Closed-time histograms: KCR1 did not affect the number of native I_f_ closed states, while KCR1 knock-down resulted in a loss of one closed state. Number of closed events (square root) were plotted against the logarithmically binned closed time durations for I_f_ alone and with exogenous KCR1 or KCR1^siRNA^ (pooled one-channel experiments only).

Direct modulation of I_f_ by KCR1 overexpression was further analysed in single-channel recordings. Notably, single-channel properties of native I_f_ in control cells more closely resembled those of heterologously expressed HCN2+KCR1 than of I_HCN2_ alone (Figure 6A, Table 2), indicating a possible modulation of HCN subunits by endogenous KCR1 in native cells. KCR1 further suppressed single-channel availability, open probability, and single-channel amplitude and conductance of native I_f_ (Figure 6A and 7A; Table 2), and shifted the voltage of half-maximal activation to more negative values (adult ventriculocytes: from −66.3±4.5 mV, n = 5, to −95.8±3.8 mV, n = 7; p<0.001, Figure 6B; neonatal ventriculocytes: from −56.4±1.3 mV, n = 12, to −81.3±3.2 mV, n = 10; p<0.0001, Figure 7B), without significantly affecting the slope factor k for adult (control: −11.9±2.1 mV, KCR1: −17.8±3.1 mV), and neonatal ventriculocytes (control: −12.1±3.9 mV, KCR1: −16.7±4.8 mV). These data indicate that KCR1 modulates single-channel behavior of I_f_ in cardiac tissue.

**Table 2:** Single-channel parameters of native I_f_ in adult and neonatal cardiomyocytes modulated by KCR1 and KCR1^siRNA^

Parameter	Adult myocyte I_f_ control	Adult myocyte I_f_ KCR1-infected	Neonatal myocyte I_f_ control	Neonatal myocyte I_f_ KCR1-infected	Neonatal myocyte I_f_ KCR1^siRNA^-infected
Open probability (%)	4.10±0.74	0.94±0.24*	9.12±1.72#	4.05±1.09*	16.3±3.24* #
Availability (%)	45.2±4.08	21.8±2.97*	41.2±8.46#	20.4±5.11*	50.2±7.55#
Mean open time (ms)	0.55±0.06	0.56±0.05	0.43±0.06	0.40±0.05	0.47±0.05
Mean closed time (ms)	1.85±0.28	2.22±0.10	2.41±0.34	3.94±0.65	2.13±0.37#
Mean first latency (ms)	53.9±6.80	62.7±4.35	40.5±5.51	46.3±7.49	28.6±4.22#
Amplitude (pA)	−0.85±0.05	−0.59±0.02*	−0.86±0.02#	−0.59±0.02*	−1.23±0.11* #
Conductance (pS)	8.81±0.25	6.10±0.26*	7.01±0.49#	4.27±0.31*	13.6±0.94* #
I_peak_ (fA)	70±9	27±2*	49±13	23±7	102±27
number of experiments	10 (6)	9 (5)	12 (5)	10 (6)	9 (5)

Modulation of single-channel parameters of native I_f_ (control) by KCR1 overexpression (KCR1-infected) and knock-down of endogenous KCR1 (KCR1^siRNA^-infected) in adult and neonatal cardiomyocytes. Holding potential −35 mV, test potential −90 mV. I_peak_ was measured from ensemble average currents. For closed time and latency analysis, only experiments containing just one detected open level were used for calculation. Numbers of experiments given in parentheses indicate number of experiments with only one channel in the patch. Pooled data are presented as mean±SEM.

* p<0.05 vs. control;

# p<0.05 vs. KCR1-infected.

While KCR1 reduced the number of HCN2 open states from four to three in the heterologous expression system (CHO cells), in which no endogenous KCR1 was detectable, KCR1 had no effect on gating kinetics of native I_f_ in cardiomyocytes. In neonatal ventriculocytes we detected only three open and three closed states (I_f_-τ_open_ values [ms]: 0.26 [58.9%], 0.87 [32.8%], 4.54 [8.3%] (n = 12) vs. I_f_+KCR1-τ_open_ values [ms]: 0.25 [47.8%], 1.22 [43.1%], 5.56 [9.1%] (n = 10); I_f_-τ_closed_ values [ms]: 0.18 [57.5%], 1.57 [33.6%], 12.7 [8.9%] (n = 5) vs. I_f_+KCR1-τ_closed_ values [ms]: 0.15 [45.1%], 1.13 [37.3%], 16.5 [17.6%] (n = 6), Figure 7C and 7D), whereas in adult ventriculocytes we observed two more closed state (I_f_-τ_open_ values [ms]: 0.23, 0.94, 4.64 (n = 7) vs. I_f_+KCR1-τ_open_ values [ms]: 0.18, 0.99, 4.59 (n = 6); I_f_-τ_closed_ values [ms]: 0.16 [60.2%], 0.99 14.7%], 1.21 [17.6%], 5.47 [5.4%], 34.68 [2.1%] (n = 6) vs. I_f_+KCR1-τ_closed_ values [ms]: 0.13 [48.3%], 0.92 [24.1%], 1.24 [17.9%], 5.76 [5.8%], 34.84 [3.9%] (n = 5), Figure 6C and 6D). These results suggest that endogenous KCR1 already modulated open kinetics of native I_f_.

### Suppression of endogenous KCR1 increases I_f_ current density, single-channel activity of native I_f_ and spontaneous beating rate

While alteration of I_f_ by KCR1 overexpression already indicated a direct interaction of HCN channels and KCR1 in native tissue, we further confirmed this finding by suppression of endogenous KCR1 using siRNA. Quantitative real-time PCR verified specific KCR1 knock-down by our siRNA in transfected CHO cells (normalized KCR1 mRNA: KCR1 100±0.09%; KCR1+pENTR/U6-KCR1^siRNA^ 34.8±0.03%; KCR1+pENTR/U6-LacZ^siRNA^ 91.1±0.08%) and neonatal cardiomyocytes (normalized KCR1 mRNA: control 100±0.07%; KCR1+pENTR/U6-KCR1^siRNA^ 31.6±0.09%; KCR1+pENTR/U6-LacZ^siRNA^ 96.5±0.1%). To evaluate the functional modulation of KCR1 knock-down we choose neonatal myocytes, because (i) these cells enable both analysis of KCR1-mediated effects on I_f_ current and spontaneous beating activity, and (ii) adult cardiomyocytes exhibit a high rate of dedifferentiation during culture for >48 h [22].

Compared to control conditions suppression of endogenous KCR1 significantly increased whole-cell I_f_ current density (6.09±0.6 pA/pF n = 11; p = 0.04 vs. control, Figure 5A, 5C and 5D). Moreover, KCR1 knock-down markedly increased the single-channel activity and altered the gating behavior of native I_f_ (Figure 7, Table 2). In contrast to KCR1 overexpression, KCR1 knock-down shifted the voltage of the half-maximal activation to more positive values (−44.5±0.9 mV, n = 15; p<0.05 vs. control; slope factor k −8.61±3.8 mV; p = n.s vs. control, Figure 7B). Consistent with our observations in experiments with enhanced and heterologeous KCR1 expression, analyses of the multi-state gating revealed the opposite effect by KCR1 knock-down on native I_f_, i.e. an increase in the number of open states and a loss of one closed-state (τ_open _values of I_f_-KCR1^siRNA^ [ms]: 0.26 [46.4%], 1.19 [10.0%], 1.26 [25.7%], 3.21 [17.9%] (n = 9); τ_closed _values of I_f_-KCR1^siRNA^ [ms]: 0.10 [79.9%], 8.38 [20.1%] (n = 5); Figure 7C and 7D).

**Figure 8:**
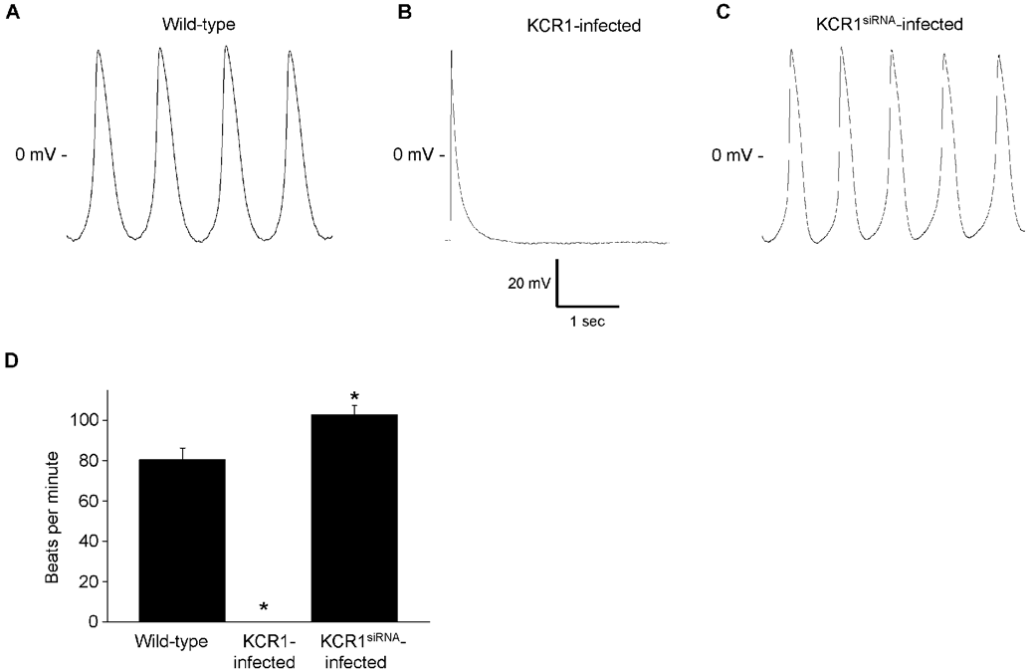
KCR1 suppresses spontaneous action potential activity. (A) Representative original recordings of spontaneous action potentials of control neonatal rat ventricular cardiomyocytes. (B) KCR1 infection suppressed spontaneous beating activity in neonatal cells. Action potential artificially induced by a depolarizing pulse in a quiescent neonatal cardiocyte. (C) KCR1^siRNA^ infection accelerated spontaneous beating activity in neonatal cells. (D) Overexpression and knock-down of endogenous KCR1 resulted in a significant (_*_, p<0.001) decrease and increase of the beating rate, respectively. For data see text.

To evaluate a possible functional modulation of KCR1 in spontaneously active tissue action potential recordings were performed in spontaneously beating neonatal cardiocytes. Control cultures beat spontaneously with a mean rate of 80.4±5.8 bpm (n = 17). Cycle length tended to vary from beat to beat (Figure 8A). Maximal diastolic potential was −56.4±3.5 mV. KCR1 overexpression entirely suppressed beating activity in neonatal cardiocytes (n = 20; maximal diastolic potential −58.6±2.7 mV; p = n.s. vs. control). Figure 8B illustrates a representative recording of an action potential that was induced artificially by a short depolarizing current pulse in a KCR1-infected myocyte. Conversely, knock-down of endogenous KCR1 by siRNA resulted in an acceleration of the beating rate to 100.2±4.6 bpm (n = 17; p<0.005 vs. control; maximal diastolic potential −55.8±3.3 mV; p = n.s. vs. controls, Figure 8C and 8D). This obvious inhibitory effect of KCR1 on action potential frequency further supports the critical contribution of I_f_ to spontaneous beating activity of neonatal cardiocytes [4] and the modulating effect of KCR1 on native I_f_ and automaticity.

## Discussion

In addition to the pore forming α-subunit Na^+^, Ca^2+^ and K^+^ channels incorporate modulatory β-subunits, as well as scaffolding proteins, chaperones, cytoskeletal elements, and Ca^2+^-sensing proteins in their higher order structures [23]–[25]. KCR1 is a membrane-associated protein with 12 putative transmembrane regions [13], [14]. In the present study KCR1 expression was demonstrated in neonatal and adult ventriculocytes. By coimmunoprecipitation KCR1 was shown to form protein complexes with HCN2. KCR1 markedly altered I_HCN_ and I_f_ whole-cell currents, single-channel activity and gating, and suppressed spontaneous action potential activity, indicating a functional interaction with HCN channels.

Heterologously expressed HCN channels result in hyperpolarization-activated inward currents with similar, however, not identical properties compared to native I_f_ [8], [9], [12]. These observations suggest that HCN subunits are modulated by additional β-subunits and regulatory proteins in native tissue. Recent studies demonstrated that HCN channels associate with several modulating proteins [26]–[31]. KCNE2 enhances the expression and accelerates activation of I_HCN_ [26]–[28]. Filamin A modifies HCN1, but not HCN2 or HCN4 channel activity and distribution on the cell membrane [29]. Moreover, TPR-containing Rab8b interacting protein [30], tamalin, S-SCAM and Mint2 scaffold proteins [31] were reported to affect HCN trafficking and, thus, I_HCN_ current density. However, since these effects are not sufficient to explain I_f_ properties in native tissue, it is likely that HCN currents are being modulated by one or more additional proteins.

Using immunoprecipitation studies we now show that KCR1 and HCN2 proteins associate. It is well known that regulatory proteins can affect both expression and gating of pore forming α-subunits. Suppression of recombinant HCN2 single-channel activity by KCR1 clearly demonstrated a functional effect of KCR1 on HCN channel behavior. More importantly, knock-down of endogenous KCR1 also profoundly modulated native I_f_ function in ventriculocytes. Loss of KCR1 lead to (i) an increase of I_f_ current density, (ii) a higher activation of I_f_ on single-channel level, and (iii) a significant elevation of the spontaneous beating rate.

Currently, two distinct ranges of single channel conductances for native I_f_ and cloned HCN channels have been reported depending on the recording technique (low conductance currents of 1–1.5 pS using a corner frequency of 80–800 Hz [17], [18], [20] vs. an approximately 10-fold higher conductance using a filtering of 2000 Hz [16], [19]), as has been discussed previously [32]. In the present study we further confirmed that current openings in our recordings indeed were produced by HCN channels as we demonstrated (i) a typical increase of channel activity by the application of cAMP to the inner side of inside-out patches, and (ii) a characteristic channel inhibition by the I_f_/HCN specific blocker ivabradine. Moreover, we could clearly show time- and voltage-dependence of HCN single-channel recordings obtained from multi-channel patches which were comparable to macroscopic I_f_/I_HCN_ currents. Given the evidence for cooperative gating between single HCN channels [20], expectedly, the sum of recordings from one-channel patches in the present study and in our previous report [16] did not result in average currents with identical kinetics compared to macroscopic I_f_/I_HCN_, emphasising that for the evaluation of I_f_/I_HCN_ averages it is essential to distinguish between recordings obtained from patches with one individual channel vs. multi-channel patches.

While differences of I_f_/I_HCN_ conductance in part might be caused by distinct recording techniques or by the expression of different HCN channel isoforms, it might also indicate HCN modulation by endogenous KCR1 within native channel complexes. Notably, in the present study single-channel parameters of native I_f_ more closely resembled those of HCN2+KCR1 than HCN2 alone (Table 1 and 2), like for instance single-channel conductance in ventriculocytes (7–9 pS). A similar single-channel conductance (7–10 pS) has been reported for recordings of I_f_ in neurons [19], [33], which also express KCR1 [13], supporting the hypothesis of potential I_f_ modulation by KCR1 in native tissue. Beside single-channel conductance a possible interaction of endogenous KCR1 and HCN was especially evident for single-channel open kinetics. While four open states could be obtained for HCN2 alone in heterologous expression, KCR1 reduced HCN2 open kinetics to only three states, which was however similar to native I_f_. Consistent with this notion, knock-down of endogenous KCR1 increased kinetics of native I_f_ to four open states and increased single-channel conductance. These observations further indicate that KCR1 functionally interacts with native I_f_ in cardiac tissue.

Interestingly, KCR1 overexpression shifted the voltage-dependence of I_f_ activation to more negative potentials, whereas KCR1 knock-down resulted in a shift of the half-maximal activation to more positive values. Low expression levels of KCR1 in sinus node cells might contribute to larger I_f_ current size and more positive voltages of mid-activation of I_f_, whereas higher expression of KCR1 in the atrioventricular node and working myocardium could explain much smaller I_f_ currents and the more negative threshold potentials of I_f_ activation in these regions. However, it should always be acknowledged that (i) the regional distribution of HCN channel isoforms (e.g. HCN4 as the predominant isoform in sinoatrial node region), (ii) the variety of heteromultimerization, and (iii) the influence of other known/unknown modulating proteins together determine the biophysical behavior of native I_f_. This might explain why single channel conductance of native I_f_ in sinoatrial node cells was not reported to be higher than in working myocardium or neurons [16], [17], [19], [33].

KCR1 inhibited single-channel activity and seemed to keep HCN channels in a deeper closed-state indicating a functional alteration of HCN channel subunits as underlying mechanism of KCR1-mediated modulation of I_f_ and cardiac automaticity. Further potential KCR1-induced regulatory mechanisms might involve alteration of HCN trafficking and cell-surface stability. Although such effects could not explain the changes of I_f_ single-channel gating, they might contribute to the observed KCR1-mediated actions and will be addressed in future studies.

KCR1 significantly suppressed spontaneous beating activity of neonatal cardiocytes, implying that KCR1 contributes to modulation of the spontaneous activity in cardiac cells. Interestingly, both KCNE2 and KCR1 originally were identified as potential regulatory subunits of HERG, the pore forming α-subunit of I_Kr_ [14], [34]. These and our observations suggest, that both proteins may have a prominent role for the balance of inward (I_f_) and outward (I_Kr_) currents involved in cardiac pacemaker activity.

Given these obvious inhibitory effects of KCR1 on HCN channels and native I_f_, alterations of KCR1 expression might cause changes in heart rate or ectopic spontaneous depolarizations in patients, though our *in-vitro* observations cannot readily be generalized to humans *in-vivo*. Interacting proteins like KCR1 inhibiting I_f_ and, thus, depressing spontaneous activity may provide a novel therapeutic target of cardiac arrhythmias.

## Materials and Methods

### Plasmids, Small Interfering RNA and Adenovirus Preparation

The expression plasmids pAdCGI-HCN2 encoding the full-length sequence of mHCN2, pCGI-Kv1.3AYA and pAdCGI have been described [4], [35]. The full-length coding sequence of rat KCR1 (kindly provided by Dr. H. Higashida, Kanazawa University, Japan) was cloned into the multiple cloning site of pAdCGI, to give pAdCGI-KCR1. The red fluorescent protein coding sequence (Red) of psDRed-Express-1 (Clontech, Palo Alto, CA) was cloned into pAdCGI-HCN2 in place of the EGFP sequence, to generate pAdCRI-HCN2. For immunoprecipitation studies, the rat KCR1 sequence was ligated in frame at the N-terminus to the three FLAG epitopes of vector p3xFLAG-CMV7.1 (Sigma GmbH, Munich), resulting in the plasmid pCFLAG^3^-KCR1.

The small interfering RNA (siRNA) nucleotide sequences for rat KCR1 (Accession no. GI 3513450) and control LacZ were as follows: 5′-GGAAACGTAGAGTTCAGTTCTC-3′ and 5′-CTACACAAATCAGCGATTT-3′ (Invitrogen), respectively. For transient transfection experiments the DNA oligonucleotides were cloned into pENTR/U6 (Invitrogen) to give pENTR/U6-KCR1^siRNA^ and pENTR/U6-LacZ^siRNA^, respectively, and for adenovirus generation into pAdHCRed to give pAdHCRed-KCR1^siRNA^. Adenovirus vectors were generated by Cre-lox recombination of purified ψ5 viral DNA and shuttle vector DNA as previously described [4], [35], [36].

### Transient Transfections

CHO-K1 cells (ATCC, American Type Culture Collection, Manassas, VA) were transfected with 1 µg/well plasmid DNA (as indicated) using Lipofectamine Plus (Life Technologies, Gaithersburg, MD) as directed by the manufacturer [37], [38]. Three days posttransfection, electrophysiological recordings, RNA isolation and immunoprecipitations from whole cell protein extracts were performed.

### Rat Cardiomyocyte Isolation and Primary Culture

Animal experiments were performed in accordance with institutional guidelines for animal use in research. Ventricular myocytes of adult rats (Sprague-Dawley, 200 to 300 g) were isolated using Langendorff apparatus as previously described [39], [40]. Neonatal cardiomyocytes of Sprague-Dawley (1–2 days old) rats were enzymatically dissociated as previously described [4]. Action potential studies were conducted on 4- to 6-day-old monolayer cultures. For whole-cell patch-clamp experiments, 3- to 5-day-old monolayer cultures were dispersed by trypsin, and re-plated at a low density to study electrically isolated cells within 2 to 8 hours [4].

### Adenovirus Infection

Infection of adult and neonatal myocytes was performed 2 h and 1–3 days after plating, respectively, at a multiplicity of infection (MOI) of 15 to 100 p.f.u. per cell.

### Immunoprecipitation

Whole protein extracts were prepared by lysing the cells in washing buffer containing 50 mM Tris (pH 7.4), 150 mM NaCl, 0.25% Triton X-100, and 5 mM NaF, supplemented with the mammalian protease inhibitors Aprotinin (Sigma). Extracts were then passed through a needle 15 times, followed by high-speed centrifugation to sediment cellular debris. 700 µg of each extract were immunoprecipitated with protein A-Sepharose-linked anti-FLAG-M2 antibodies (Sigma). A set of identical control experiments was performed using beads not linked to antibody (normal A-Sepharose, Sigma). Immunoprecipitates were washed three times with lysis buffer followed by resuspension in SDS sample buffer (0.23 M Tris, pH 6.4, 10% glycerol, 0.33% SDS, 3.3 mM DTT), sonication, and electrophoresis on a 7.5% SDS-PAGE. The gel was transferred to Hybond ECL membrane (Amersham) for Western blotting with an anti-HCN2 antibody (Alomone Labs, Israel).

### Single-cell RT-PCR analysis

cDNA synthesis and PCR reaction from two to three isolated single cells were performed using the OneStep RT-PCR Kit (QIAGEN, Hilden, Germany) as directed by the manufacturer with sense- and antisense primer specific for KCR1 (sense: 5′-GAC GAG ATC TTC CAC CTG C-3′; antisense: 5′-TAG AAG TTG CCA ACA CTG AAG-3′; amplifying 218 base pairs and spanning one intron) and GAPDH (sense: 5′-GGT CGG TGT GAA CGG ATT TG-3′; antisense: 5′-GTG AGC CCC AGC CTT CTC CAT-3′; amplifying 318 base pairs). Amplification was performed in a Thermocycler gradient (Eppendorf, Hamburg, Germany), starting with 95°C for 15 minutes to deactivate the Reverse Transcriptase and activate the Taq polymerase. The program consisted of 50 cycles (Denaturation: 95°C for 30 seconds; Annealing: 59±3°C gradient for 45 seconds; Elongation: 72°C for 1 minute) with a final elongation step at 72°C for 10 minutes. Samples were analyzed on 1.5% agarose gels, stained with ethidium bromide and documented on a BioDocAnalyze 2.0 (Biometra, Göttingen, Germany).

### Preparation of sinus and atrioventricular node

Fresh hearts from adult rats (Sprague-Dawley, 200 to 300 g) and adult pigs (freshly received from slaughterhouse) were dissected. To locate the sinoatrial node we used three intersection lines: the sulcus terminalis, the lateral border of the superior vena cava, and the superior border of the superior vena cava or the right auricle. The atrioventricular node was prepared according to the anatomic landmarks of the triangle of *Koch* in the right atrium: the coronary sinus ostium, the membranous septum, and the septal/posterior comissure of the tricuspid valve. Finally the left and right atria, left and right ventricular free wall and ventricular septum were dissected. RNA was directly isolated from these fresh samples.

### RNA isolation and Quantitative Real-time PCR

Total RNA was extracted from neonatal rat cardiomyocytes, and from cardiac tissue of adult rat and pig using the RNeasy kit (Qiagen), and first strand cDNA was reverse transcribed with Omniscript Reverse Transcription Kit (Qiagen) as described previously [41]. Quantitative real-time PCR was performed on a LightCycler™ 2.0 instrument using FastStart DNA Master^Plus^ SYBR Green I (Roche Diagnostics). Gene-specific primers were the following: KCR 1 for rat samples: forward-primer: 5′-TTC AGG AAG ATA CAG CCC AGA-3′, backward-primer: 5′-GGG TTG GAA ATA CTG CTA GGG-3′; KCR1 for pig samples: forward-primer: 5′-GAC GAG ATC TTC CAC CTG C-3′, backward-primer: 5′-TAG AAG TTG CCA ACA CTG AAG-3′. GAPDH (glyceraldehyde-3-phosphate dehydrogenase) and SDHA (succinate dehydrogenase complex, subunit A, flavoprotein) expression of each sample were used as endogenous controls (GAPDH - primers: 5′-GGT CGG TGT GAA CGG ATT TG-3′ and 5′-GTG AGC CCC AGC CTT CTC CAT-3′, GenBank accession No. NM_017008; SDHA-primers: 5′-TGG GAA CAA GAG GGC ATC TG-3′ and 5′-CCA CCA CTG CAT CAA ATT CAT G-3′, GenBank accession No. NM_130428) [42]. Quantitative real-time PCR was carried out under the following cycling conditions: stage 1, 95°C for 10 minutes (rep 1); stage 2, 95°C for 10 s, 61°C for 5 s, and 72°C for 10s (rep 38). Analysis of the PCR curves was performed with the second derivate maximum method of the LightCycler software [41], [43], [44]. All sample measurements were repeated at least three times and results are given as mean±SEM.

### Electrophysiology and Data Analysis

Experiments were carried out using standard microelectrode whole-cell and cell-attached patch-clamp techniques with an Axopatch 200B amplifier and Digidata 1200 interface (Axon instruments, Foster City, CA, USA) at room temperature (21 to 23°C) [35], [45], [46]. Whole-cell and single-channel recordings were done as previously described, if not otherwise indicated [4], [16], [41], [47]. For inside-out recordings both the pipette and bath solution were composed of (mM): KCl 160 mM, MgCl_2_ 1 mM, HEPES 10 mM, EGTA 1 mM; pH 7.4 with KOH. In some experiments 8-bromo-adenosine 3′,5′-cyclic monophosphate (8Br-cAMP, 1 mM) (Sigma) or ivabradine (50 µM; kindly provided by the Institut de Recherches Servier, Suresnes, France) were added to the bath solution, as indicated. In KCR1-infected myocytes action potentials were initiated by short depolarizing current pulses (2 ms, 500–800 pA).

Single-channel analysis were done using custom software as previously reported [16], [45], [47]. Linear leak and capacity currents were digitally subtracted using the average currents of non-active sweeps. For detailed gating analysis idealized currents were analyzed in 150 ms steps recorded at continuous pulse mode (total recording 20×150 ms). Closed-time and first-latency analyzes were carried out only in one-channel patches. The open probability (defined as the relative occupancy of the open state during active sweeps), the availability (fraction of sweeps containing at least one channel opening), and I_peak_ (the peak ensemble average current, obtained visually) were calculated from single-channel and multi-channel patches with a maximum of three active channels in one patch. Single-channel amplitudes were determined by direct measurements of fully resolved openings (conductance-calculation) or as the maximum of Gaussian fits on amplitude histograms. n, the number of the channels in the patch, was defined as the maximum current amplitude observed, divided by the unitary current. Peak current was corrected by division through n. The availability was corrected by the square root method: (1-availibiltiy_corrected_) is the n^th^ root of (1-availibiltiy_uncorrected_). The corrected open probability was calculated on the basis of the corrected number of active sweeps, i.e. total open time divided by (n×availability_corrected_×number of test pulses×pulse length). Time constants of open time (τ_open_) and closed time histograms (τ_closed_) were obtained by multi-exponential maximum likelihood estimates (*MLE*) on open and closed time distributions (shown here as logarithmic binned histograms from pooled data) is given by the “best fit” method [21]. Comparison of gating kinetics was performed as previously described [16].

The availability or fractions of active sweeps were used for calculation of voltage-dependent activation. The voltage-dependence of activation was analyzed using the Boltzmann function: Y = Y_max_/{1+e ^(V 0.5-V/k)^}, where V_0.5_ is the voltage of half-maximal activation and k is the slope factor [45]. Pooled data are presented as mean±SEM. An unpaired two-tailed *t* test was used for statistical examinations. Probability values of *P*<0.05 were deemed significant. One-way ANOVA was calculated for multiple comparisons and considered significant for *P*<0.05.
