# Correction: K^+^ Channel Regulator KCR1 Suppresses Heart Rhythm by Modulating the Pacemaker Current I_f_

**DOI:** 10.1371/annotation/ff27c2a4-7732-4c32-88da-c412f1cced0c

**Published:** 2012-08-08

**Authors:** Guido Michels, Fikret Er, Ismail F. Khan, Jeannette Endres-Becker, Mathias C. Brandt, Natig Gassanov, David C. Johns, Uta C. Hoppe

The authors note that two baseline traces in Figure 6A (right panel) appeared incorrectly. Due to a copy and paste error while processing the original current traces for the preparation of the figure, the first and third traces and the eleventh and twelfth traces were duplicated. This error does not affect scientific results or the conclusions of the article. The authors apologise for this error and are providing the corrected figure via this Correction.

The revised version of Figure 1 can be seen here: http://www.plosone.org/corrections/pone.0001511.006.cn.tif [^] 

